# Anti-tumor activity of the TGF-β receptor kinase inhibitor galunisertib (LY2157299 monohydrate) in patient-derived tumor xenografts

**DOI:** 10.1007/s13402-014-0210-8

**Published:** 2015-01-09

**Authors:** Armin Maier, Anne-Lise Peille, Vincent Vuaroqueaux, Michael Lahn

**Affiliations:** 1 In Vitro Screening, Oncotest GmbH, Am Flughafen 12-14, 79108 Freiburg, Germany; 2 Molecular Biology, Oncotest GmbH, Am Flughafen 12-14, 79108 Freiburg, Germany; 3 Biomaker Development, Oncotest GmbH, Am Flughafen 12-14, 79108 Freiburg, Germany; 4 Early Phase Clinical Investigation, Eli Lilly and Company, Indianapolis, 46285 IN USA; 5 6820 Wisconsin Avenue, Condo 8008, Bethesda, MD 20815 USA

**Keywords:** Anti-tumor activity, Patient-derived xenografts, TGF-β, Galunisertib, Gene expression

## Abstract

**Purpose:**

The transforming growth factor-beta (TGF-β) signaling pathway is known to play a critical role in promoting tumor growth. Consequently, blocking this pathway has been found to inhibit tumor growth. In order to achieve an optimal anti-tumor effect, however, it remains to be established whether blocking the TGF-β signaling pathway alone is sufficient, or whether the tumor microenvironment plays an additional, possibly synergistic, role.

**Methods:**

To investigate the relevance of blocking TGF-β signaling in tumor cells within the context of their respective tissue microenvironments, we treated a panel of patient-derived xenografts (PDX) with the selective TGF-β receptor kinase inhibitor LY2157299 monohydrate (galunisertib) and assessed both the in vitro and in vivo effects.

**Results:**

Galunisertib was found to inhibit the growth in an in vitro clonogenic assay in 6.3 % (5/79) of the examined PDX. Evaluation of the expression profiles of a number of genes, representing both canonical and non-canonical TGF-β signaling pathways, revealed that most PDX exhibited expression changes affecting TGF-β downstream signaling. Next, we subjected 13 of the PDX to an in vivo assessment and, by doing so, observed distinct response patterns. These results suggest that, next to intrinsic, also extrinsic or microenvironmental factors can affect galunisertib response. pSMAD2 protein expression and *TGF-βRI* mRNA expression levels were found to correlate with the in vivo galunisertib effects.

**Conclusions:**

From our data we conclude that intrinsic, tumor-dependent TGF-β signaling does not fully explain the anti-tumor effect of galunisertib. Hence, in vivo xenograft models may be more appropriate than in vitro clonogenic assays to assess the anti-tumor activity of TGF-β inhibitors such as galunisertib.

**Electronic supplementary material:**

The online version of this article (doi:10.1007/s13402-014-0210-8) contains supplementary material, which is available to authorized users.

## Introduction

The transforming growth factor beta (TGF-β) signaling pathway plays a pleiotropic role in both normal and tumor tissues, including tumor-stroma interactions [1, 2]. The canonical TGF-β signaling pathway becomes activated when 1 of the 3 ligands (TGF-β1, TGF-β2, TGF-β3) binds to the TGF-β receptor II (TGF-βRII), which subsequently heterodimerizes with the TGF-β receptor I (TGF-βRI or ALK5) and transphosphorylates the kinase domains of both receptors. This phosphorylation step leads to a recruitment and phosphorylation of SMAD2 and SMAD3 (pSMAD2 and pSMAD3). Next, this complex initiates the canonical or SMAD-dependent signaling cascade leading to nuclear translocation and downstream gene transcription [3]. In addition to the canonical signaling pathway, other activation pathways (non-canonical pathways) have been described, but these are less understood [4]. The non-canonical or non-SMAD-dependent activation of the TGF-β pathway includes signaling via jun N-terminal kinase (JNK), p38 MAPK, ERK or MEKK.

In the past, several small molecule inhibitors targeting the TGF-βRI serine/threonine kinase activity have been developed, including LY2157299 monohydrate (galunisertib) [5], which has been found to inhibit pSMAD2 expression in different tumor models [6, 7]. Galunisertib is now being investigated in a clinical trials and has very recently been shown to elicit anti-tumor effects in patients with glioblastoma or hepatocellular carcinoma [8, 9]. Since only a few TGF-β inhibitors are currently being studied in clinical trials, the development of appropriate preclinical models is considered imperative in order to reliably establish the mechanisms of action of TGF-β inhibitors and to specifically direct new drug screens.

In traditional models such as xenografts with established tumor cell lines or in vitro cell viability studies, galunisertib has shown moderate anti-tumor activity [10, 11]. Here, we used patient-derived xenografts (PDX) instead of established tumor-derived cell lines [12, 13]. In contrast to these cell lines, primary patient-derived cells generally retain their original phenotype [14, 15]. Initially, primary patient-derived cells were used to assess the effects of cytotoxic agents [16] but, more recently, these cells have also been found to be useful for characterizing anti-tumor activities of cytostatic or immunomodulatory agents [17–19]. It has also been noted that the effects of anti-tumor drugs in PDX-based clonogenic assays correlated well with clinical responses observed in patients with various solid cancers [12, 20, 21]. Therefore, we hypothesized that PDX models might be useful for testing both the in vitro and in vivo effects of galunisertib in different primary tumor cell types and, as such, to delineate the roles of both intrinsic and extrinsic activities of TGF-β signaling in the respective responses in these models.

## Materials and methods

### Small molecule TGF-β receptor kinase inhibitor

The small molecule LY2157299 monohydrate (galunisertib), targeting TGF-βRI serine/threonine kinase activity, was provided by Eli Lilly and Company, Indianapolis, USA. Galunisertib was tested at concentrations ranging from 0.03 to 10.0 μM. Selected tumor xenografts were re-tested at higher concentrations ranging from 1.0 to 80.0 μM. Stock solutions of the compound were prepared in DMSO at 3.0 or 24.0 mM, respectively, and small aliquots were stored at −20 °C in the dark. Final dilutions were prepared in Iscove’s Modified Dulbecco’s Medium (IMDM, Life Technologies, Carlsbad, CA) immediately prior to use.

### In vitro clonogenic assays on patient-derived xenograft samples

After obtaining the informed consent from patients and approvals from local ethics review boards, patient-derived xenograft (PDX) samples were derived from tumors subcutaneously growing as xenografts in NMRI nu/nu mice [13, 22] purchased from Elevage Janvier, France or Taconic Europe, Denmark. Details of the test procedure have been previously described [21]. Briefly, solid tumor xenografts were removed from mice under sterile conditions, mechanically disaggregated and subsequently incubated in an enzyme cocktail consisting of collagenase type IV (41 U/ml), DNase I (125 U/ml), hyaluronidase type III (100 U/ml) and dispase II (1.0 U/ml) in RPMI-1640 medium at 37 °C for 45–60 min. Single cells were passed through sieves of 200 and 50 μm mesh size and washed twice with sterile PBS. The percentage of viable cells was determined in a hemocytometer using trypan blue exclusion staining.

The tumor clonogenic assays were performed according to a modified soft agar assay introduced by Hamburger & Salmon [23]. Each test well contained three layers of equal volumes: two layers of semi-solid medium (bottom and top layer) and one layer of medium supernatent with or without test compound. The bottom layer consisted of IMDM, supplemented with 20 % (*v/v*) fetal calf serum (FCS, Sigma, St Louis, MO), 0.01 % (*w/v*) gentamicin (Life Technologies, Carlsbad, CA) and 0.75 % (*w/v*) agar (BD Biosciences, San Jose, CA). Cells were seeded at a final density of 7.5 × 10^4^ to 2 × 10^5^ cells/ml using the same culture medium, supplemented with 0.4 % (*w/v*) agar, and plated onto the bottom layer. The test compound was added by continuous exposure (drug overlay) in culture medium. Cultures were incubated at 37 °C and 7.5 % CO_2_ in a humidified atmosphere for 7–20 days and monitored closely for colony growth using an inverted microscope. Within this period, colonies were formed with a diameter > 50 μm. At the time of maximum colony formation, counts were performed using an automatic image analysis system (OMNICON 3600, Biosys GmbH, Germany) after staining vital colonies for 24 h prior to evaluation with a sterile aqueous solution of 2-(4-iodophenyl)-3-(4-nitrophenyl)-5-phenyltetrazolium chloride (1 mg/ml, 100 μl/well) [24]. Additionally, the viability of the colonies was determined using a CellTiter-Glo® viability assay (Promega, Madison, WI), as an equivalent to colony formation, and luminescence was measured using an EnVision® Xcite Multilabel Reader (Perkin Elmer, Waltham, MA) to quantify the amount of metabolically active and, thus, viable cells. All assays were performed in a standardized manner and the efficacy of galunisertib was assessed in relation to an untreated control only containing cells and the solvent (DMSO at 0.3 %). The efficacy of galunisertib was rated based on concentration-responses as: inhibition (T/C ≤ 75 %), no response (75 % < T/C < 125 %) or stimulation (T/C ≥ 125 %).

### In vivo assays on patient-derived xenografts

Patient-derived xenografts (PDX) were established from primary patient material as described above. Cell line-derived xenografts were established from cells harvested from in vitro culture. Xenografts were subcutaneously grown in nude mice through serial passage and randomized after reaching tumor volumes of approximately 72–120 mm^3^.

Galunisertib was prepared as a suspension in 1 % NaCMC, 0.5 % SLS, 0.05 % Antifoam, 0.085 % PVP C-30, and administered twice daily for 14 days at a dose of 75 mg/kg orally by gavage (12 mice per group). The control group received an identical volume of the same mix without galunisertib. The tumor load was determined by caliper measurement twice weekly and the absolute tumor volume [mm^3^] was calculated according to the formula: a [mm] × b^2^ [mm^2^] × 0.5, where (a) is the largest diameter and (b) is the perpendicular diameter of the tumor representing an idealized ellipsoid. The relative volume of an individual tumor on day x (RTV_x_) was calculated by dividing the absolute volume [mm^3^] of the respective tumor on day x (T_x_) by the absolute volume of the same tumor on the day of randomization, i.e., on day 0 (T_0_), multiplied by 100: RTV_x_ [%] = T_x_/T_0_ × 100. Group median RTVs were used for drawing tumor growth curves and for treatment evaluation. The tumor growth response was expressed quantitatively by the Area Between the Curves (ABC), comparing the Area Under the Curve (AUC) of tumor growth curves of the control group with the AUC of the group treated with galunisertib according to the formula below.

$$ \boldsymbol{A}\boldsymbol{B}\boldsymbol{C}\ \left(\%\right)=\frac{\boldsymbol{AU}{\boldsymbol{C}}_{\boldsymbol{control}}-\boldsymbol{AU}{\boldsymbol{C}}_{\boldsymbol{treatment}}}{\boldsymbol{AU}{\boldsymbol{C}}_{\boldsymbol{control}}}*100 $$

Using this formula, positive ABC values represent tumor growth curves below the control group and indicate growth inhibition. Tumor responses were classified according to ABC values of < −20 % = growth stimulation, −20 % < ABC < 20 % = no change, > 20 % = growth inhibition, compared to the control. All studies were performed in agreement with German animal welfare acts.

### RNA isolation

For each untreated xenograft model, tissues were pooled from 4 different mice. Total RNA was extracted from frozen samples using the “mirVana miRNA Isolation kit” (Ambion, Carlsbad, CA) according to the manufacturer’s instructions. Genomic DNA was removed using the “RNase-free DNase Set” (Qiagen, Hilden, Germany). The quality of the RNA preparations was controlled using a Bioanalyzer (Agilent Technologies, Palo Alto, CA). Only RNA samples with an RNA integrity number (RIN) > 6.5 were used for gene expression profiling purposes (see below).

### DNA isolation

For DNA isolation, snap frozen samples from untreated tumors were digested with proteinase K at 55 °C overnight and lysates were digested with RNase A (Qiagen). Next, DNAs were extracted using phenol:chloroform:isoamylalcohol and precipitated with ethanol. DNA pellets were washed and resuspended in TElow buffer (Tris 10 mM pH8, EDTA 0.1 mM pH8). The integrity of the DNA samples was checked after 1.3 % agarose gel electrophoresis, and the purity of the DNA samples was determined using a NanoDrop 2000 system (Thermo Scientific, Waltham, MA).

### Gene expression profiling

Total RNAs were submitted to AROS Applied Biotechnologies (Aarhus, Denmark) or DNA vision for analysis on Affymetrix HGU133 plus 2.0 gene expression arrays. First- and second-strand synthesis, biotin labeling, fragmentation and hybridization were performed according to Affymetrix protocols. Evaluation and normalization of the Affymetrix GeneChip Data were performed in the “R” (version 2.15.3) statistical computing environment. The hybridizations were normalized using the gcRMA (gc robust multichip averaging) method from Bioconductor to obtain summary expression values for each probe set. One probeset for each gene was chosen according to Li et al. [25]. Gene expression levels were analyzed on a logarithmic scale and were expressed in arbitrary units (U). Affymetrix expression values < 6 U were considered as background.

### Gene mutation analyses

The mutation status of key cancer genes was assessed in all samples using mass array sequencing panels from Sequenom, Inc. (OncoCarta panels I, II and III) and then confirmed by Sanger sequencing of individual exons or whole exome sequencing. Moreover, 64/79 PDX samples were profiled by whole exome sequencing. Exonic regions from Oncotest DNA samples were targeted using Agilent SureSelect Human All Exon kits 38 MB (60 samples) or 51 MB (4 samples). Enriched genomic DNA was sequenced using an Illumina HiSeq-2000 platform in 100 bp paired-end (PE) reads and an expected coverage of ~80×. To remove the mouse stroma content, PE reads that mapped better on the mouse (mm10) than on the human (hg19) genome were discarded from the human mapped read dataset (based on the Burrows-Wheeler Alignment mapping score) using PicardTools. Variants were detected by independently using 3 different variant callers: the GATK’s UnifiedGenotyper, the combination of Samtools mpileup and bcftools caller, and the Freebayes caller. Only variants identified with all 3 tools, showing a minimum number of variant-supporting reads of 3 and a minimum variant frequency of 5 %, were further analyzed.

### SNP profiling

The Affymetrix Genome-Wide Human SNP Array 6.0 with 1.8 million genetic markers, including more than 906,600 single nucleotide polymorphisms (SNPs) and more than 946,000 probes for the detection of copy number variation (CNV), was employed using a standard protocol recommended by the manufacturer. According to the Affymetrix guidelines, contrast Quality Control (QC) and Median Absolute Pairwise Difference (MAPD) thresholds were set at values > 0.4 and 0.35, respectively.

CNVs were identified using the Affymetrix Genotyping Console^™^ v4.1 and the PICNIC software provided by the Cancer Genome Project from the Welcome Trust Sanger Institute [26].

### Western blot analyses

Native tumor lysates were prepared for Western blotting as previously described [17]. Briefly, 4 parts of untreated PDX samples were homogenized using a Tissue Lyser (Qiagen) in cell lysis buffer supplemented with Tris pH7.4 20 mM (Roth, Karlsruhe, Germany), NaCl 100 mM (Merck, Darmstadt, Germany), EDTA 1 mM (Sigma-Aldrich), NP40 1 % (Sigma-Aldrich), deoxycholate 0.5 % (Sigma-Aldrich), Na_4_O_7_P_2_ 10H_2_O 10 mM (Sigma-Aldrich), Na_3_VO_4_ 2 mM (Sigma-Aldrich), NaF 20 mM (Sigma-Aldrich), PMSF 100 μM (Sigma-Aldrich), Benzonase 50 U/ml, and a protease inhibitor mix 1× (Roche, Mannheim, Germany). Lysates were cleared and protein concentrations measured (Protein Assay, Bio-Rad, Berkley, CA). After polyacrylamide gel electrophoresis and membrane transfer, the resulting membranes were incubated with primary antibodies (anti- TGF-β1 clone 56E4, Cat #3709, rabbit mAb, Cell Signaling, Beverly, CA; anti-Smad2: clone D43B4, Cat #5339, rabbit mAb, Cell Signaling; anti-pSmad2 (ser465/467): clone 138D4, Cat #3108, rabbit mAb, Cell Signaling; anti-GAPDH: clone 14C10, Cat #2118, rabbit mAb, Cell Signaling). The binding of primary antibodies was detected using a secondary HRP-coupled antibody (goat anti-rabbit IgG (H + L)-HRP conjugate, 170-6515-Biorad), followed by incubation with an ECL Western blotting detection reagent (GE Healthcare Cat # RPN2106, Little Chalfont, United Kingdom) and visualization using a GE Healthcare Image Quant LAS 4000 CCD camera system (GE Healthcare 28-9558-10). Quantification of protein expression was performed by subtracting the intensity of the signal of GAPDH from the intensity of the signal of the protein of interest, and was carried out using Image J software. The calculated quantities of the respective proteins are expressed in arbitrary units (AU). The Oncotest tumor lysate pool containing a mixture of 380 different PDX lysates was used as a normalization control to minimize plate-to-plate variability.

## Results

### TGF-β inhibitor galunisertib variably impairs clonogenic growth of tumor-derived xenografts

The TGF-β inhibitor galunisertib was evaluated in different PDX and cell line-derived xenografts (CDX) in different test series using a clonogenic assay. The test panel consisted of 11 different tumor types, including glioblastoma, melanoma, colon, gastric, liver, non-small cell lung (adenocarcinoma, squamous cell carcinoma and large cell lung carcinoma, NSCLC), small cell lung, mammary, ovary, pancreas, and renal cell cancers (Table 1). In addition, established cell lines derived from hematologic malignancies including leukemia, lymphoma and myeloma were tested. Initial screens with diverse numbers of tumor types have often been used to assess the range of anti-tumor efficacy for compounds in order to understand their differential activity [27–29]. The majority of PDX in our current study were obtained from metastatic tumor lesions (42/79, 53 %) and were poorly differentiated. Most tumors were obtained from males (46/79; 58 %) and the mean age of the patients was 52 years (median age 55 years, range 11–82 years). The anti-tumor effects were recorded as inhibition of colony formation in relation to untreated controls (T/C values, see materials and methods). Although doses above 10 μM are expected to be associated with unspecific activity of galunisertib [6], we used higher concentrations to gain insight into anti-tumor effects beyond the pharmacologically targeted concentration.

**Table 1:** Main characteristics of 79 patient-derived xenografts (PDX) and cell line-derived xenografts (CDX) (solid and hematologic), which were tested against galunisertib in different test series

Histologic type	Origin (PDX / CDX)	Tumor designation	Pathological stage at time of xenograft implantation	Histology classification	Origin of tumor lesion	Origin of organ	Differentiation	Patient age	Gender
Glioblastoma	PDX	CNXF 498	WHO Grade IV	glioblastoma	not known	Brain	poorly differentiated	62	male
Colon	PDX	CXF 1103	T3N1M1	adenocarcinoma	metastasis	Skin (peritoneum)	poorly differentiated	55	male
Colon	PDX	CXF 158	T2N0M0	adenocarcinoma	primary	Colon	moderately differentiated	68	female
Colon	PDX	CXF 1784	M1	adenocarcinoma	metastasis	Liver	moderately differentiated	65	male
Colon	PDX	CXF 260	TxN2Mx	adenocarcinoma	primary	Colon	poorly differentiated	68	female
Colon	PDX	CXF 269	TxN2Mx	colorectal adenocarcinoma	primary	Colon	poorly differentiated	56	male
Colon	PDX	CXF 280	M1	adenocarcinoma	metastasis	Skin	poorly differentiated	55	female
Colon	PDX	CXF 742	M1	adenocarcinoma	metastasis	Liver (mesenterium)	poorly differentiated	66	male
Colon	PDX	CXF 975	T3N1M0	adenocarcinoma	primary	Colon	moderately differentiated	44	female
Colon	CDX	CXF-HCT-116	not known	colon carcinoma	not known	Colon	poorly differentiated	not known	male
Gastric	PDX	GXF 1172	T4N3M1	adenocarcinoma	metastasis	Pleura	poorly differentiated	49	male
Gastric	PDX	GXF 251	T2N0M0	adenocarcinoma	primary	Stomach	poorly differentiated	60	male
Liver	PDX	LIXF 575	not known	hepatocellular carcinoma	primary	Liver	well differentiated	66	male
Liver	CDX	LIXF Hep-G2	not known	hepatocellular carcinoma	not known	not known	poorly differentiated	15	male
Lung, NSC	PDX	LXFA 1012	T2N0Mx	adenocarcinoma	primary	Lung	poorly differentiated	58	male
Lung, NSC	PDX	LXFA 1041	T4NxMx	adenocarcinoma	metastasis	Pleura	well differentiated	60	male
Lung, NSC	PDX	LXFA 1584	T4N1Mx	adenosquamous carcinoma	primary	Lung	poorly differentiated	53	male
Lung, NSC	PDX	LXFA 1647	M1	adenocarcinoma	metastasis	adrenal gland	poorly differentiated	53	male
Lung, NSC	PDX	LXFA 297	T2N2Mx	adenocarcinoma	metastasis	Lymph Node	poorly differentiated	46	male
Lung, NSC	PDX	LXFA 526	N3M1	adenocarcinoma	metastasis	Lymph Node	poorly differentiated	58	male
Lung, NSC	PDX	LXFA 677	T1N1Mx	adenocarcinoma	primary	Lung	poorly differentiated	62	male
Lung, NSC	PDX	LXFA 737	T3N2Mx	adenocarcinoma	primary	Lung	moderately differentiated	56	male
Lung, NSC	PDX	LXFA 749	T2N1-2 Mx	adenocarcinoma	primary	Lung	poorly differentiated	72	male
Lung, NSC	PDX	LXFA 983	T2N0M1	adenocarcinoma	metastasis	Brain	poorly differentiated	59	male
Lung, NSC	CDX	LXFA CALU-6	not known	adenocarcinoma	not known	not known	poorly differentiated	61	female
Lung, NSC	PDX	LXFE 1422	T2N3Mx	squamous cell carcinoma	primary	Lung	poorly differentiated	38	male
Lung, NSC	PDX	LXFE 211	M1 skin	squamous cell carcinoma	metastasis	Skin	poorly differentiated	58	male
Lung, NSC	PDX	LXFE 397	T1N0Mx	squamous cell carcinoma	primary	Lung	poorly differentiated	56	male
Lung, NSC	PDX	LXFL 1121	T2N1M0	large cell carcinoma	primary	Lung	poorly differentiated	56	female
Lung, NSC	PDX	LXFL 1176	TxN2Mx	large cell carcinoma	metastasis	Lymph Node	moderately differentiated	65	male
Lung, NSC	PDX	LXFL 529	T3N1M0	large cell carcinoma	primary	Lung	poorly differentiated	34	female
Lung, NSC	PDX	LXFL 625	T3N1M0	large cell carcinoma	primary	Lung	poorly differentiated	39	female
Lung, SC	PDX	LXFS 615	M1	small cell anaplastic carcinoma	metastasis	Bone Marrow	poorly differentiated	56	female
Lung, SC	PDX	LXFS 650	N1	small cell carcinoma	metastasis	Lymph Node	poorly differentiated	64	male
Breast	PDX	MAXF 1162	T4N0Mx	adenocarcinoma	primary	breast	poorly differentiated	55	female
Breast	PDX	MAXF 1322	M1	adenocarcinoma	metastasis	Brain	poorly differentiated	49	female
Breast	PDX	MAXF 1384	M1	adenocarcinoma	metastasis	Brain	poorly differentiated	32	female
Breast	PDX	MAXF 401	M1	adenocarcinoma	metastasis	Lung	well differentiated	51	female
Breast	PDX	MAXF 449	T3N1M0	invasive ductal carcinoma	primary	breast	poorly differentiated	53	female
Breast	PDX	MAXF 574	not known	invasive ductal carcinoma	not known	not known	poorly differentiated	not known	female
Breast	PDX	MAXF 583	M1	adenocarcinoma	metastasis	Lung	moderately differentiated	50	female
Breast	PDX	MAXF 857	T4NxM0	adenocarcinoma	primary	breast	poorly differentiated	60	female
Breast	PDX	MAXF MX1	not known	not known	not known	not known	poorly differentiated	29	female
Breast	CDX	MAXF-MDA-MB-231	not known	invasive ductal carcinoma	not known	not known	poorly differentiated	51	female
Melanoma	PDX	MEXF 1341	M1	melanoma	metastasis	Lung	not known	35	female
Melanoma	PDX	MEXF 1539	N1	melanoma	metastasis	Lymph Node	not known	36	male
Melanoma	PDX	MEXF 1732	N1	melanoma	metastasis	Lymph Node	not known	33	male
Melanoma	PDX	MEXF 1737	N1	melanoma	metastasis	Lymph Node	not known	62	female
Melanoma	PDX	MEXF 1765	N1	melanoma	metastasis	Lymph Node	not known	49	male
Melanoma	PDX	MEXF 1792	N1	melanoma	metastasis	Lymph Node	not known	60	male
Melanoma	PDX	MEXF 1829	M1	melanoma	metastasis	Lymph Node	not known	41	female
Melanoma	PDX	MEXF 1870	pM1 (SKI), R1	melanoma	metastasis	Skin	not known	63	female
Melanoma	PDX	MEXF 2090	not known	melanoma	primary	Skin	not known	not known	not known
Melanoma	PDX	MEXF 2095	not known	melanoma	primary	Skin	not known	not known	not known
Melanoma	PDX	MEXF 2106	not known	melanoma	primary	Skin	not known	not known	not known
Melanoma	PDX	MEXF 274	N1	melanoma	metastasis	Lymph Node	not known	51	male
Melanoma	PDX	MEXF 276	N1	melanoma	metastasis	Lymph Node	not known	22	male
Melanoma	PDX	MEXF 394	not known	melanoma	metastasis	Skin	not known	70	male
Melanoma	PDX	MEXF 462	M1	melanoma	metastasis	Skin	not known	63	male
Melanoma	PDX	MEXF 514	N1	melanoma	metastasis	Lymph Node	not known	40	female
Melanoma	PDX	MEXF 520	M1	melanoma	metastasis	Skin	not known	41	male
Melanoma	PDX	MEXF 535	N1	melanoma	metastasis	Lymph Node	not known	55	male
Melanoma	PDX	MEXF 622	M1	melanoma	metastasis	Lung	not known	68	male
Melanoma	PDX	MEXF 666	M1	melanoma	metastasis	Skin	not known	67	male
Melanoma	PDX	MEXF 672	N1	melanoma	metastasis	Lymph Node	not known	25	male
Melanoma	PDX	MEXF 989	M1	melanoma	metastasis	Lymph Node	not known	32	male
Ovary	PDX	OVXF 1353	N1	serous adenocarcinoma	metastasis	Lymph Node	poorly differentiated	41	female
Ovary	PDX	OVXF 550	not known	serous adenocarcinoma	not known	not known	poorly differentiated	not known	female
Ovary	PDX	OVXF 899	T3N0M0	serous adenocarcinoma	primary	ovary	well differentiated	76	female
Pancreas	PDX	PAXF 1657	M1	adenocarcinoma	metastasis	Lung	moderately differentiated	60	female
Pancreas	PDX	PAXF 736	M1	adenocarcinoma	recurrence	Pancreas	poorly differentiated	65	male
Kidney	PDX	RXF 1220	T3M1	hypernephroma	metastasis	Lung	not known	59	male
Kidney	PDX	RXF 1393	T4N0Mx G2	hypernephroma	metastasis	Lung	not known	56	male
Kidney	PDX	RXF 631	M1	hypernephroma	metastasis	Lung	not known	54	male
Hematologic tumors									
Leukemia	CDX	LEXF-K-562	not known	chronic myelocytic leukemia	not known	not known	poorly differentiated	53	female
Lymphoma	CDX	LYXF-MYLA	not known	T cell cutaneous	not known	not known	well differentiated	82	male
Lymphoma	CDX	LYXF-RAJI	not known	B lymphocyte	not known	not known	poorly differentiated	11	male
Lymphoma	CDX	LYXF-U-937	not known	histiocystic lymphoma	not known	not known	poorly differentiated	37	male
Myeloma	CDX	MMXF-L-363	not known	multiple myeloma	not known	not known	poorly differentiated	36	female

**Fig. 1:**
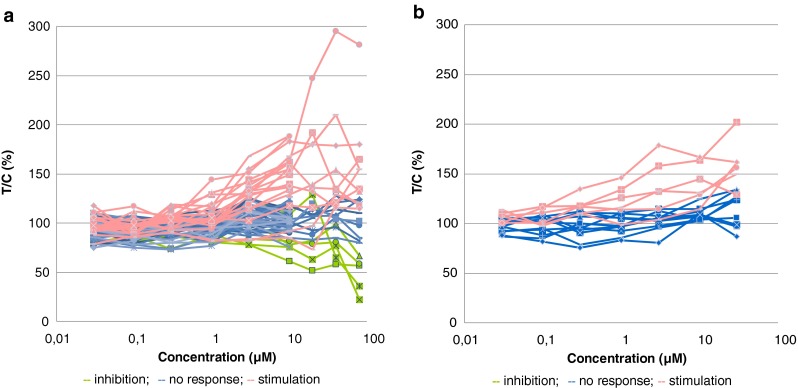
Responses of human PDX samples to galunisertib (LY2157299) treatment assessed in an in vitro anchorage-independent growth assay. **a**) Image analysis-based evaluation of colony formation revealed inhibition (*green lines*), no response (*blue lines*) or stimulation (*pink lines*) of colony formation across different tumor xenografts. **b**) Viability-based evaluation of colony formation revealed no response (*blue lines*) or stimulation (*pink lines*) of colony formation in patient-derived melanoma xenografts. The efficacy of galunisertib was rated based on concentration-response as: inhibition (T/C ≤ 75 %), no response (75 % < T/C < 125 %), or stimulation (T/C ≥ 125 %)

The efficacy of galunisertib was assessed in a first series of experiments in a panel of 66 tumor models. The compound inhibited colony growth in 1/66 samples (1.5 %) in a concentration-dependent manner when tested up to 10 μM. Growth stimulation was observed in 15/66 (22.7 %) of the samples and no response was seen in 50/66 (75.8 %) of the samples. When tested up to 80 μM, galunisertib inhibited colony growth in 5/24 (20.8 %) selected samples. Stimulation of colony formation was observed in 10/24 (41.7 %) of these samples and no response was found in 9/24 (37.5 %) of the samples (Fig. 1a). The most sensitive xenograft samples were CXF 742 (colon cancer), LXFS 650 and LXFE 1422 (small cell lung cancer), and the hematologic xenograft samples LYXF MYLA (T-ALL) and LYXF RAJI (Burkitt’s lymphoma). No correlation between responses and histopathological characteristics of the different samples was observed (Fig. 1a).

We also assessed galunisertib in a second series of experiments in panel of melanoma PDX (Fig. 1b). This melanoma panel was chosen because of previous reports suggesting that TGF-β1 signaling is an autocrine activation pathway for tumor cell growth in melanoma [30–32]. We found that galunisertib did not have any inhibitory effect in this panel of melanoma xenografts, i.e., when tested up to 10 μM galunisertib elicited no response in 12/17 (70.6 %) of the samples, whereas stimulation of colony formation was observed in 5/17 (29.4 %) of the samples. At a higher concentration (30 μM), no response was observed in 11/17 (64.7 %) of the samples, whereas stimulation of colony formation was observed in 6/17 (35.3 %) of the samples.

### Molecular characteristics of tumor models investigated ex vivo

Because of the limited inhibitory effects observed in the clonogenic assays, we set out to investigate whether the canonical TGF-β signaling pathway is altered in these PDX. To this end, we assessed the expression level and mutation status of genes associated with the canonical (i.e., the *TGF-β1*, *TGF-β2*, *SMAD2*, *SMAD3*, *SMAD4*, *SMAD7*, *TGF-βRI* and *TGF-βRII* genes) as well as the non-canonical (i.e., the *MAPK* and *AKT* genes) TGF-β signaling pathways. The aims were (i) to characterize the molecular profiles of these pathways and (ii) to evaluate whether the respective genes predicted drug sensitivity. We also investigated the expression of two proteins that were previously reported to be associated with TGF-β -mediated drug resistance, i.e., TP53 and MED12 [33].

The copy numbers of the *TGF-β1, TGF-β2, TGF-βR1* and *TGF-βR2* genes were assessed in 70/79 PDX samples. No major rearrangements were observed. Two samples showed mutations in *TGF-β1*: CXF 260, A350V and MEXF 989, S138L. Two samples showed mutations in *TGF-β2*: CXF 269, S365R, and CXF 260, P387H. Three samples showed mutations in *TGF-βR1*: MAXF 401, E242D, CXF 260, A125V and LXFA 737, E111K. All these mutations were found in the non-responder group. *TGF-βR2* was mutated in 4 samples, including one from the group that showed growth inhibition (LXFE 1422,153X [HGVS nomenclature for frameshift]), one from the group that showed growth stimulation (LXFA 1041, N384T) and two from the group that showed no response (LXFA 526, 153X and CXF 1103, 128 fs, C393F). No associations were found between the mutation status of the PDX samples and the responses to galunisertib (data not shown).

**Fig. 2:**
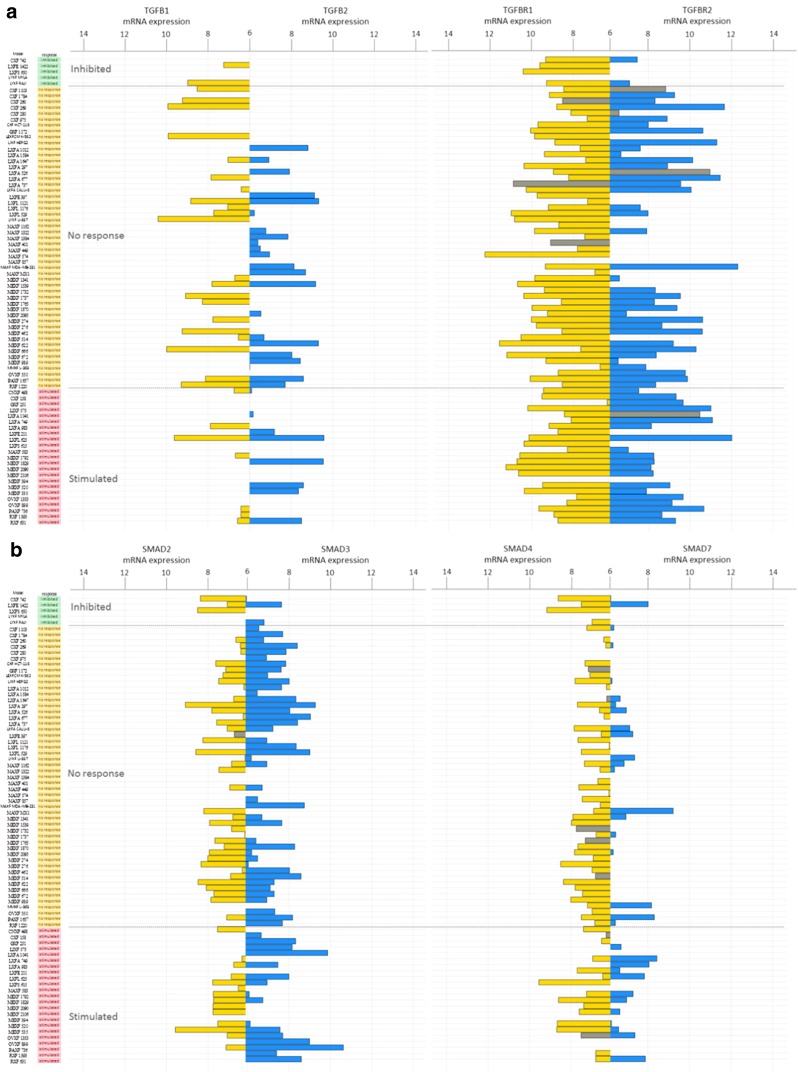
Bar plot representing mRNA expression of genes associated with the canonical TGF-β pathway using Affymetrix HGU133 plus2.0 arrays: (**a**) *TGF-β1* (*yellow*) and *TGF-β2* (*blue*), and *TGF-βR1* (*yellow*) and *TGF-βR2* (*blue*) and (**b**) *SMAD2* (*yellow*) and *SMAD3* (*blue*), and *SMAD4* (*yellow*) and *SMAD7* (*blue*). The PDX samples are ranked by their respective responses to galunisertib treatment, i.e., inhibited (*green*), no response (*yellow*) or stimulated (*pink*). Grey bars indicate PDX mutated for the gene of interest. For each gene, one probe-set was selected according to Li et al. [25]. mRNA expression levels < 6 were considered as background and are not represented in this figure

Next, we set out to investigate the mRNA expression levels of the *TGF-β1, TGF-β2, TGF-βR1* and *TGF-βR2* genes in 77/79 PDX samples (Fig. 2, Panel a). Heterogeneous mRNA expression levels were found for these 4 genes in the samples tested, and no significant associations were found between the mRNA expression levels and the responses to galunisertib. Despite this variability, however, some general trends were observed: (i) the expression levels of the *TGF-β1* and *TGF-β2* genes were generally low or undetectable, (ii) the *TGF-βR1* gene was well expressed in most of the samples tested, whereas the *TGF-βR2* gene was expressed at a low level or undetectable in at least some of the samples (Fig. 2, panel a).

We also assessed the status of the TGF-β1 downstream canonical activation pathway (SMAD-dependent activation). No major gene copy number alterations were found in the samples tested, except in LXFA 749 (deletion of *SMAD3*) and in LIXF 575 (deletion of *SMAD4*), both from the “stimulated” group. Sequence analysis revealed mutations in *SMAD1* (CXF 260, A262V, not shown) and *SMAD2* (LXFE 397, S287C). No mutations were found in *SMAD3*. *SMAD4* was the most frequently mutated gene with 9 mutations in samples from the “no response” (7/9) and the “stimulated” (2/9) groups. *SMAD6* was mutated in 3 samples of the “inhibited” and the “no response” groups (LXFS 650, L192P; CXF 260, D359G and LXFA 1012, P323L; not shown). *SMAD7* was mutated in CXF 260 (A159V and D113G) and MAXF 449 (R131C). All SMAD genes (*SMAD1, 2, 3, 4, 6 and 7*) showed heterogeneous expression patterns, but these patterns were not found to be associated with a response to galunisertib (Fig. 2). Of note, *SMAD7* expression was nearly absent in most samples, whereas *SMAD2, SMAD3* and *SMAD4* were expressed at similar levels, with similar minimum to maximum ranges (Fig. 2b), and variable patterns in most samples.

**Fig. 3:**
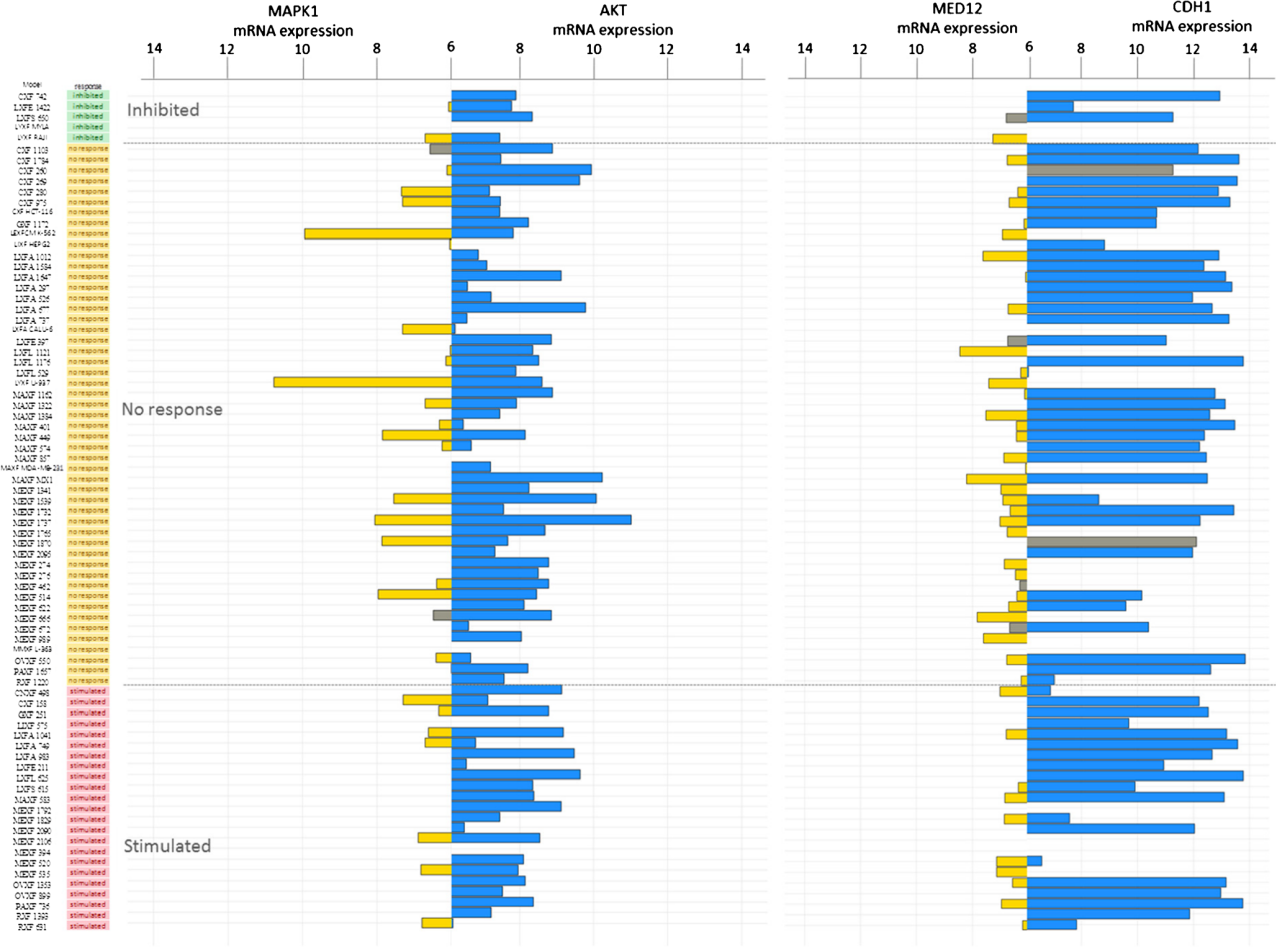
Bar plot representing mRNA expression of genes associated with the non-canonical TGF-**β** pathway using Affymetrix HGU133 plus2.0 arrays: *MAPK1* (*yellow*) and *AKT1* (*blue*), and *MED12* (*yellow*) and *CDH1* (*blue*). The PDX samples are ranked by their respective responses to galunisertib treatment, i.e., inhibited (*green*), no response (*yellow*) or stimulated (*pink*). Grey bars indicate PDX mutated for the gene of interest. For each gene, one probe-set was selected according to Li et al. [25]. mRNA expression levels < 6 were considered as background and are not represented in this figure

Subsequently, we subjected some non-canonical or SMAD-independent genes [4, 33] to genomic and transcriptomic analyses. No major genomic alterations in the *AKT1 gene* were discovered, and *AKT1* mRNA expression was detected in most of the samples tested (range 5.5-11 Units [U], mean 7.9 U). There was no significant association with respons to galunisertib. The *MAPK1* gene was found to be expressed in 30 % of the samples investigated, and no associations between its expression and galunisertib responses were observed. E-cadherin (*CDH1*) expression levels were observed in most of the samples tested (Fig. 3).

We next investigated the status of some genes presumed to be associated with drug resistance in relation to TGF-β signaling (Fig. 3). No overt *MED12* gene copy number gains were detected. In 21 samples, however, loss of one *MED12* gene copy was noted without any observable alteration in mRNA expression. *MED12* gene mutations were detected in 8 samples, of which 6 exhibited mRNA expression levels that were below the detection threshold. None of the alterations observed were found to be associated with a galunisertib response. Of note, *TP*53 mutations were frequently encountered in most of the samples tested (not shown), but these mutations could not significantly be associated with responses to galunisertib (Kruskal-Wallis *p-*value = 0.13).

**Fig. 4:**
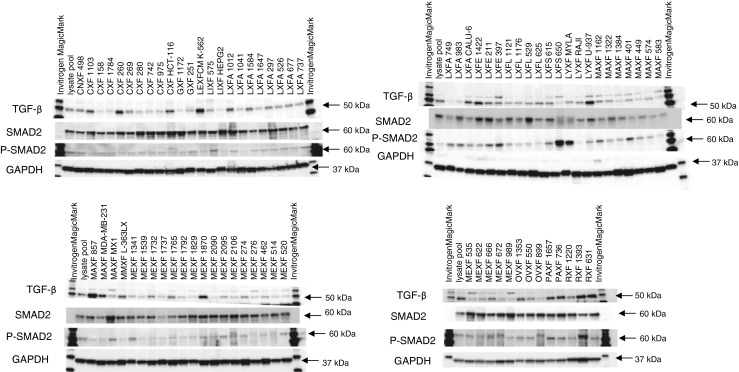
Western blots showing TGF-β1, SMAD2 and pSMAD2 protein expression levels in 79 untreated PDX samples. GAPDH was used as loading control. The Oncotest lysate pool (mix of protein from 380 PDX and CDX) was used in each Western blot as a normalization control

Based on the altered profiles observed in the TGF-β signaling cascade, we decided to further assess the expression of the pSMAD2 protein in order to substantiate the activated status of the TGF-β signaling pathway (Fig. 4) in the PDX samples before treatment. Most samples tested indeed showed pSMAD2 expression, and in some cases this expression was observed in spite of the fact that the TGF-βR1 protein levels were either low or undetectable.

### Galunisertib elicits in vivo anti-tumor activity

**Fig. 5:**
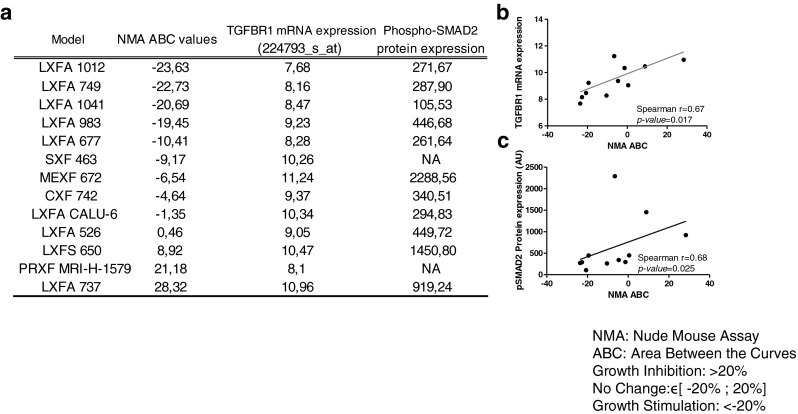
Galunisertib activities in 13 PDX samples and its correlations with *TGF-βRI* mRNA and pSMAD2 protein expression. (**a**) The in vivo efficacies were evaluated by ABC (see materials and methods) values and compared to *TGF-βRI* mRNA expression (Affymetrix HGU133 Plus 2.0) and pSMAD2 protein expression (Western blotting; see Fig. 4) levels. (**b**) Correlation between *TGF-βRI* mRNA expression levels and ABC values (%). (**c**) Correlation between pSMAD2 protein expression levels and ABC values. *P* values ≤ 0.05 are considered significant

From the 79 samples included in this study, 13 were selected for in vivo analyses. We used ABC calculations to assess the efficacy of galunisertib treatment. By doing so, we found that the ABC values ranked from −23.63 % (stimulation of tumor growth) to 28.32 % (inhibition of tumor growth) (Fig. 5a). The mRNA expression levels of genes involved in the TGF-β pathway were compared to the calculated ABC values (Fig. 5a and b). Tumor growth inhibition was seen in 2 of 13 (15.4 %) selected samples (i.e., lung cancer PDX LXFA 737 and prostate cancer PDX PRXF MRIH 1579). No changes were seen in 8/13 (61.5 %) samples. In 3/13 (23.1 %) samples, tumor growth stimulation was observed. Next, we assessed a possible association between the in vivo responses observed and the concomitant *TGF-β1* and *SMAD2* gene expression levels, or activation of the TGF-β signaling pathway by measuring pSMAD2 protein levels (Fig. 5a and c). Although the *TGF-β1* and *SMAD2* gene expression levels were not associated with the in vivo response, high *TGF-βRI* transcript levels and pSMAD2 protein levels were found to be correlated with the in vivo anti-tumor responses (Spearman correlation 0.67, *p*-value = 0.017 and Spearman correlation 0.68, *p*-value = 0.025, respectively).

## Discussion

In the present study, we observed in vitro and in vivo differences between the anti-tumor activities of the TGF-β receptor kinase inhibitor galunisertib. We also observed a lack of broad activity across the tumor panel tested, which is in stark contrast to other mostly cytotoxic anti-cancer agents. These observations are unusual for anti-cancer agents and, given the activity of galunisertib observed in patients [8], we hypothesized that the tissue microenvironment might play a critical role in supporting TGF-β-dependent tumor growth. Our current observations can be explained in several ways. First, the activity of galunisertib in the tumor clonogenic assay may be dependent on its selectivity. Galunisertib is known to selectively target TGF-βRI (ALK5) at concentrations up to 1 μM [6, 34]. Concentrations above 10 μM are not considered to be given to humans because of the possible toxic implications of this drug [35, 36]. In our current study, however, we used concentrations up to 80 μM in order to assess its effects beyond the pharmacological range. By doing so, we found no overt differences between the higher and the lower concentrations. Thus, the selectivity of the drug appeared to have little or no effect in this study. Only 5 PDX showed small inhibitory effects, not related to a specific tumor type. None of the other tumors responded to galunisertib, including melanomas, which are presumed to grow using a TGF-β-dependent autocrine loop. We grouped the galunisertib responses into three categories: “inhibited”, “no response” or “stimulated”. Second, we set out to understand why the majority of the PDX either exhibited “no response” or a “stimulated” response in vivo. Therefore, we assessed whether either the canonical or the non-canonical TGF-β1signaling pathway was intact. Canonical, SMAD-dependent TGF-β signaling was found to be defective in 30 % of the PDX samples. From this result we conclude that galunisertib may have acted on kinases for which it was not designed, resulting in unspecific activity in the clonogenic assays. *TGF-βR1* and *TGF-βR2* transcripts were detected in most of the PDX samples, whereas *TGF-β1* and *TGF-β2* transcripts were only detected at a low level and in half of the PDX. In such PDX samples autocrine loops may be active, in spite of the fact that the *TGF-β1* and *TGF-β2* levels were low. The *SMAD1*, *SMAD2* and *SMAD4* genes were all expressed. Only the *SMAD4* gene was found to be frequently mutated, while the others were rarely affected. Noteworthy, inhibitory effects of galunisertib were observed in those samples that expressed the *TGF-βR1* and *SMAD4* genes. Hence, the responses could not be traced back to a specific or intact signaling pathway, either canonical or non-canonical. Third, based on the clonogenic assay, the anti-tumor activity of galunisertib or other agents targeting the tumor microenvironment are underestimated because such agents will not show cell killing, but will arrest tumors by inhibition of cell migration and invasion [37–39]. It is also possible that galunisertib targets signals that are not tumor cell-dependent, such as immune- or other microenvironment signals, as has been shown in a syngeneic mouse model in which a monoclonal antibody against TGF-βRII showed anti-tumor activity [40]. This anti-tumor effect appeared to be dependent on the presence of specific immune cells such as CD8-positive T-cells and NK cells. We also tested galunisertib in vivo in 13 of the PDX samples. Two of these samples (LXFA 737 and PRXF MRIH 1579) showed growth inhibition (Fig. 5, Fig. S1, S2, Table S1 and S2). Given the presence of residual NK cell functions in nude mice, we assume that in these two models blocking of TGF-β-dependent signaling has facilitated NK activity against tumor cells. In addition, we noted that the PDX samples investigated contained variable amounts of human tumor cells and murine stroma components, which may contain a fibroblast-rich microenvironment [41] or genomically instable tumor cells [42]. Although the amounts of stroma in the individual samples did not directly correlate with the response rates in vivo, we found that the presence of stroma not only affects the response rate in general, but also that the in vivo response patterns correlated better with the molecular characteristics of the PDX models (i.e., pSMAD protein and *TGF-βR1* mRNA expression).

In conclusion, it appears that models that reflect the normal physiological condition, including all aspects of the microenvironment, are more useful to assess TGF-β1 inhibitors. In absence of such models, results from such experiments must be interpreted with caution.

## Electronic supplementary material

Below is the link to the electronic supplementary material.

ESM 1 (DOCX 48 kb)

ESM 2 (DOCX 36 kb)

ESM 3 (PPTX 217 kb)

ESM 4 (PPTX 124 kb)
