# Supplementary material for: Anti-tumor activity of the TGF-β receptor kinase inhibitor galunisertib (LY2157299 monohydrate) in patient-derived tumor xenografts
Source: Cell Oncol (Dordr). 2015 Jan 9;38(2):131–44. doi: 10.1007/s13402-014-0210-8 (PMC4412926; doi:10.1007/s13402-014-0210-8)
Supplement: Supplementary file 1 — (DOCX 48 kb) [file 13402_2014_210_MOESM1_ESM.docx]

**Table S1:** Ex vivo efficacy of galunisertib in a diverse set of human tumor xenografts of different histotypes as determined using a clonogenic assay using image analysis as read-out (phase 1). Efficacy of galunisertib was rated based on concentration-response as inhibition (T/C ≤75%), no response (75% < T/C <125%), or stimulation (T/C ≥125%).

|  |  |  |  | Test/control (%) at drug concentration (µM) | | | |  |  |  |
| --- | --- | --- | --- | --- | --- | --- | --- | --- | --- | --- |
| Tumor model | 0,03 | 0,1 | 0,3 | 1 | 3 | 10 | 20 | 40 | 80 | effect |
| CXF 742 | 81 | 87 | 74 | 88 | 78 | 61 | 52 | 58 | 57 | inhibition |
| LXFE 1422 | 93 | 94 | 88 | 86 | 102 | 78 | 82 | 99 | 67 | inhibition |
| LXFS 650 | 107 | 82 | 87 | 81 | 78 | 76 | 63 | 77 | 22 | inhibition |
| LYXF MYLA |  |  |  | 92 | 125 | 109 | 129 | 65 | 36 | inhibition |
| LYXF RAJI |  |  |  | 81 | 84 | 82 | 79 | 81 | 59 | inhibition |
| CXF 1103 | 85 | 92 | 100 | 99 | 96 | 100 |  |  |  | no response |
| CXF 1784 | 97 | 98 | 109 | 104 | 106 | 117 |  |  |  | no response |
| CXF 260 | 102 | 97 | 97 | 92 | 125 | 115 |  |  |  | no response |
| CXF 269 | 101 | 89 | 108 | 95 | 100 | 117 |  |  |  | no response |
| CXF 280 | 79 | 80 | 86 | 95 | 118 | 102 |  |  |  | no response |
| CXF 975 | 108 | 101 | 91 | 102 | 101 | 96 |  |  |  | no response |
| CXF HCT-116 | 80 | 83 | 90 | 99 | 125 | 111 |  |  |  | no response |
| GXF 1172 | 93 | 93 | 109 | 113 | 119 | 108 |  |  |  | no response |
| LEXF K-562 | 92 | 81 | 101 | 93 | 91 | 90 |  |  |  | no response |
| LIXF Hep-G2 | 99 | 100 | 101 | 103 | 118 | 115 |  |  |  | no response |
| LXFA 1012 | 89 | 85 | 95 | 92 | 110 | 121 |  |  |  | no response |
| LXFA 1584 | 108 | 94 | 107 | 105 | 112 | 117 | 109 | 114 | 110 | no response |
| LXFA 297 | 94 | 94 | 92 | 100 | 95 | 112 |  |  |  | no response |
| LXFA 526 | 86 | 89 | 94 | 108 | 112 | 97 |  |  |  | no response |
| LXFA 677 | 93 | 90 | 102 | 103 | 100 | 102 |  |  |  | no response |
| LXFA 737 | 98 | 88 | 90 | 98 | 108 | 120 | 107 | 128 | 119 | no response |
| LXFA CALU-6 | 106 | 107 | 103 | 94 | 93 | 101 | 93 | 96 | 82 | no response |
| LXFE 397 | 86 | 107 | 99 | 100 | 92 | 94 |  |  |  | no response |
| LXFL 1121 | 87 | 93 | 103 | 104 | 126 | 111 |  |  |  | no response |
| LXFL 1176 | 91 | 84 | 101 | 97 | 102 | 96 | 105 | 121 | 124 | no response |
| LXFL 1647 | 93 | 91 | 89 | 81 | 103 | 105 |  |  |  | no response |
| LXFL 529 | 93 | 84 | 95 | 92 | 87 | 99 | 114 | 108 | 124 | no response |
| LYXF U-937 | 85 | 89 | 88 | 92 | 120 | 106 |  |  |  | no response |
| MAXF 1162 | 91 | 95 | 86 | 93 | 111 | 109 |  |  |  | no response |
| MAXF 1322 | 107 | 90 | 103 | 85 | 98 | 124 |  |  |  | no response |
| MAXF 1384 | 93 | 91 | 99 | 111 | 110 | 119 |  |  |  | no response |
| MAXF 401 | 108 | 92 | 104 | 96 | 92 | 93 | 88 | 106 | 98 | no response |
| MAXF 449 | 84 | 77 | 91 | 85 | 118 | 104 |  |  |  | no response |
| MAXF 574 | 101 | 102 | 99 | 110 | 98 | 104 | 107 | 108 | 85 | no response |
| MAXF 857 | 103 | 107 | 88 | 107 | 88 | 85 | 83 | 85 | 80 | no response |
| MAXF MDA-MB-231 | 83 | 78 | 74 | 86 | 105 | 122 |  |  |  | no response |
| MAXF MX1 | 110 | 104 | 103 | 109 | 101 | 97 | 120 | 104 | 102 | no response |
| MEXF 1341 | 102 | 89 | 109 | 103 | 103 | 106 |  |  |  | no response |
| MEXF 1539 | 86 | 91 | 89 | 90 | 115 | 101 |  |  |  | no response |
| MEXF 1765 | 79 | 75 | 73 | 77 | 94 | 76 |  |  |  | no response |
| MEXF 276 | 94 | 84 | 97 | 103 | 100 | 92 |  |  |  | no response |
| MEXF 462 | 93 | 86 | 83 | 95 | 102 | 101 |  |  |  | no response |
| MEXF 514 | 101 | 93 | 95 | 106 | 99 | 84 |  |  |  | no response |
| MEXF 672 | 86 | 78 | 80 | 81 | 124 | 105 |  |  |  | no response |
| MMXF L-363 | 75 | 82 | 99 | 83 | 119 | 112 |  |  |  | no response |
| OVXF 550 | 96 | 83 | 93 | 111 | 113 | 102 |  |  |  | no response |
| PAXF 1657 | 99 | 98 | 94 | 95 | 97 | 102 |  |  |  | no response |
| RXF 1220 | 93 | 89 | 80 | 89 | 110 | 106 |  |  |  | no response |
| CNXF 498 | 105 | 88 | 114 | 129 | 130 | 137 |  |  |  | stimulation |
| CXF 158 | 96 | 106 | 93 | 94 | 138 | 163 | 247 | 295 | 281 | stimulation |
| GXF 251 | 96 | 89 | 95 | 117 | 144 | 162 |  |  |  | stimulation |
| LIXF 575 | 113 | 90 | 119 | 117 | 168 | 189 |  |  |  | stimulation |
| LXFA 1041 | 79 | 87 | 90 | 83 | 82 | 92 | 97 | 129 | 121 | stimulation |
| LXFA 749 | 118 | 89 | 118 | 110 | 155 | 183 | 180 | 179 | 180 | stimulation |
| LXFA 983 | 93 | 97 | 101 | 104 | 134 | 161 | 136 | 135 | 118 | stimulation |
| LXFE 211 | 103 | 99 | 95 | 102 | 114 | 129 | 139 | 154 | 132 | stimulation |
| LXFL 625 | 92 | 95 | 108 | 100 | 133 | 149 | 192 | 134 | 165 | stimulation |
| LXFS 615 | 95 | 98 | 104 | 98 | 115 | 134 |  |  |  | stimulation |
| MAXF 583 | 111 | 117 | 102 | 144 | 151 | 188 |  |  |  | stimulation |
| MEXF 1792 | 103 | 99 | 106 | 98 | 128 | 140 |  |  |  | stimulation |
| MEXF 535 | 88 | 86 | 91 | 82 | 84 | 85 | 73 | 118 | 155 | stimulation |
| OVXF 1353 | 107 | 91 | 108 | 109 | 130 | 168 | 180 | 210 | 155 | stimulation |
| OVXF 899 | 106 | 102 | 107 | 120 | 133 | 138 | 132 | 116 | 115 | stimulation |
| PAXF 736 | 96 | 96 | 106 | 102 | 119 | 156 |  |  |  | stimulation |
| RXF 1393 | 93 | 112 | 107 | 131 | 142 | 155 |  |  |  | stimulation |
| RXF 631 | 98 | 98 | 103 | 105 | 103 | 118 | 116 | 121 | 135 | stimulation |
