# Supplementary material for: Anti-tumor activity of the TGF-β receptor kinase inhibitor galunisertib (LY2157299 monohydrate) in patient-derived tumor xenografts
Source: Cell Oncol (Dordr). 2015 Jan 9;38(2):131–44. doi: 10.1007/s13402-014-0210-8 (PMC4412926; doi:10.1007/s13402-014-0210-8)
Supplement: Supplementary file 2 — (DOCX 36 kb) [file 13402_2014_210_MOESM2_ESM.docx]

**Table S2:** Ex vivo efficacy of galunisertib in human melanoma xenografts as determined using a clonogenic assay using cell viability as read-out (phase 2). Efficacy of galunisertib was rated based on concentration-response as inhibition (T/C ≤75%), no response (75% < T/C <125%), or stimulation (T/C ≥125%).

|  |  |  |  | Test/control (%) at drug concentration (µM) | | | |  |
| --- | --- | --- | --- | --- | --- | --- | --- | --- |
| Tumor model | 0,03 | 0,1 | 0,3 | 1 | 3 | 10 | 30 | effect |
| MEXF 1732 | 102 | 104 | 107 | 106 | 104 | 108 | 98 | no response |
| MEXF 1737 | 93 | 94 | 96 | 93 | 98 | 104 | 124 | no response |
| MEXF 1765 | 104 | 107 | 112 | 105 | 103 | 107 | 99 | no response |
| MEXF 1870 | 103 | 101 | 111 | 110 | 108 | 108 | 97 | no response |
| MEXF 2095 | 97 | 88 | 95 | 111 | 115 | 115 | 124 | no response |
| MEXF 274 | 92 | 95 | 79 | 86 | 96 | 103 | 127 | no response |
| MEXF 462 | 87 | 85 | 100 | 97 | 106 | 125 | 134 | no response |
| MEXF 622 | 88 | 82 | 76 | 83 | 81 | 110 | 87 | no response |
| MEXF 666 | 107 | 101 | 100 | 98 | 113 | 104 | 106 | no response |
| MEXF 672 | 97 | 109 | 94 | 97 | 106 | 112 | 134 | no response |
| MEXF 989 | 106 | 106 | 91 | 97 | 105 | 109 | 99 | no response |
| MEXF 1829 | 101 | 101 | 118 | 126 | 132 | 145 | 129 | stimulation |
| MEXF 2090 | 108 | 111 | 117 | 114 | 115 | 129 | 156 | stimulation |
| MEXF 2106 | 110 | 117 | 118 | 134 | 158 | 164 | 202 | stimulation |
| MEXF 394 | 107 | 99 | 110 | 99 | 104 | 115 | 161 | stimulation |
| MEXF 520 | 114 | 98 | 110 | 116 | 133 | 131 | 150 | stimulation |
| MEXF 535 | 101 | 116 | 135 | 146 | 179 | 167 | 162 | stimulation |
