# Supplementary figures and images for: Anti-tumor activity of the TGF-β receptor kinase inhibitor galunisertib (LY2157299 monohydrate) in patient-derived tumor xenografts
Source: Cell Oncol (Dordr). 2015 Jan 9;38(2):131–44. doi: 10.1007/s13402-014-0210-8 (PMC4412926; doi:10.1007/s13402-014-0210-8)

## Slide 1
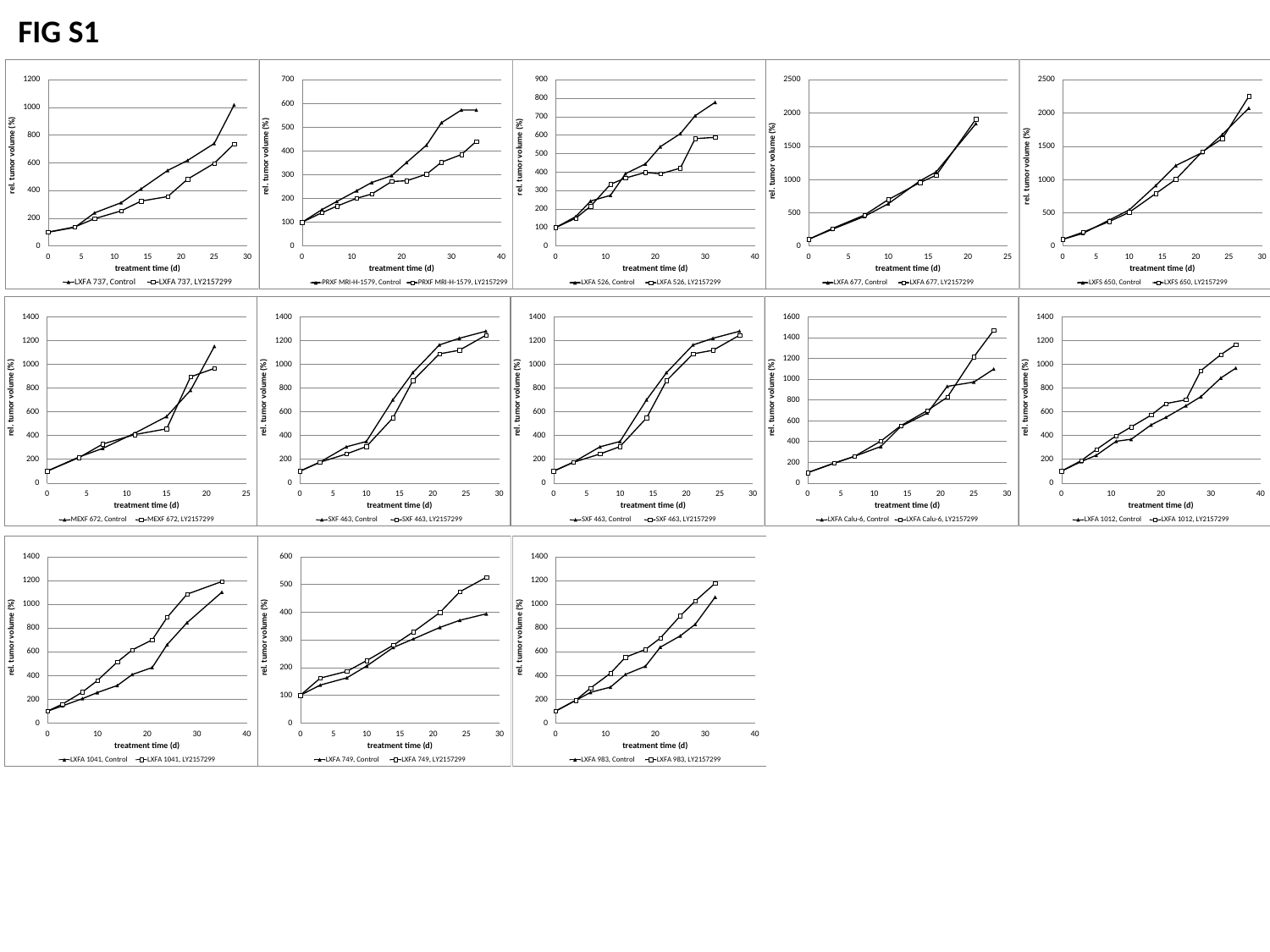

FIG S1

Supplement: Supplementary file 3 — (PPTX 217 kb) [file 13402_2014_210_MOESM3_ESM.pptx]

## Slide 1
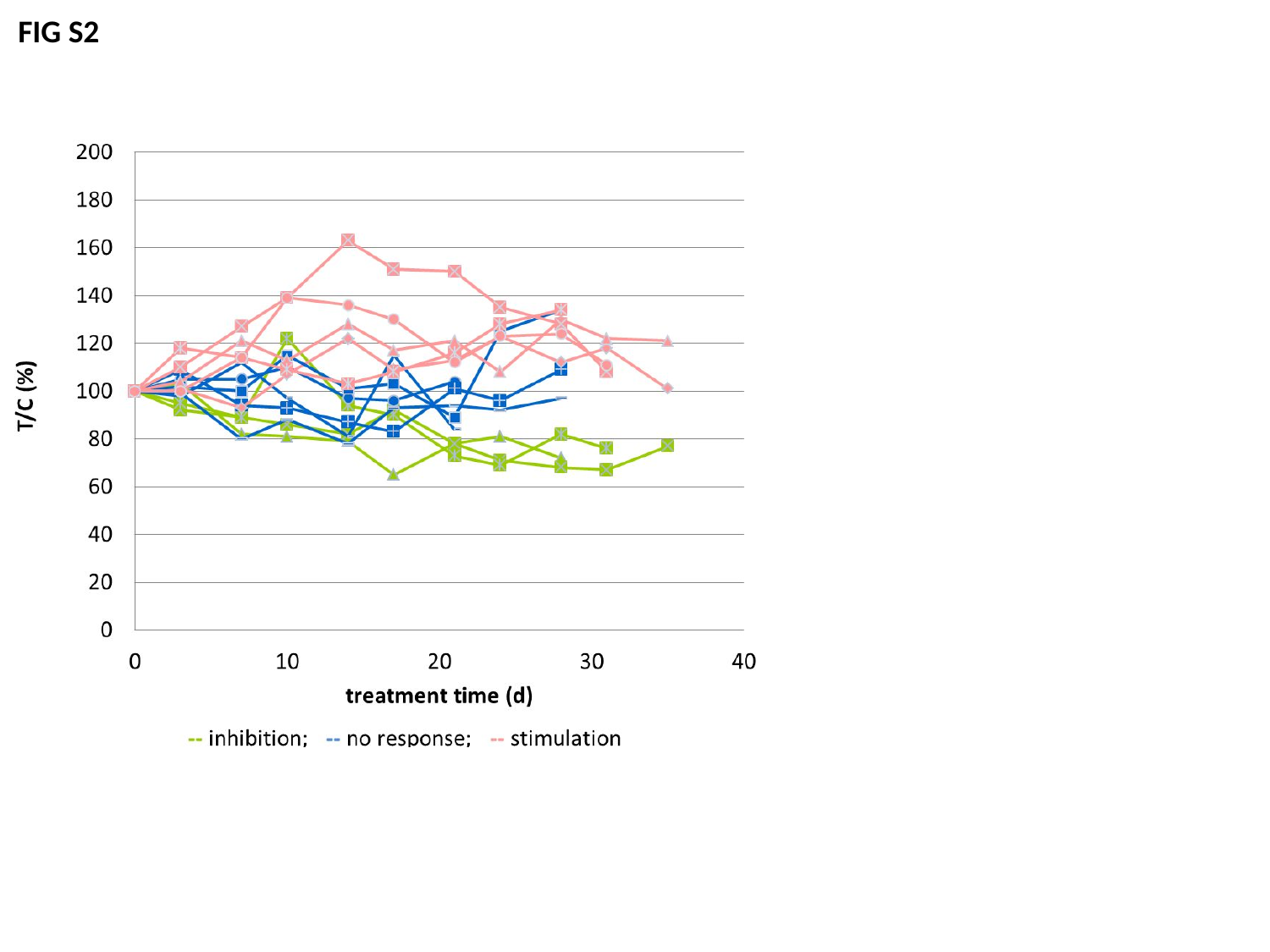

FIG S2

Supplement: Supplementary file 4 — (PPTX 124 kb) [file 13402_2014_210_MOESM4_ESM.pptx]
